# Intravenous S-ketamine’s analgesic efficacy in third molar surgery. A randomized placebo-controlled double-blind clinical trial

**DOI:** 10.1177/20494637231222327

**Published:** 2023-12-15

**Authors:** Lars B Eriksson, Torsten Gordh, Rolf Karlsten, Riccardo LoMartire, Andreas Thor, Åke Tegelberg

**Affiliations:** 1 Department of Surgical Sciences, 8097 Uppsala University, Uppsala, Sweden; 2 Centre for Clinical Research, 8097 Uppsala University, Falun, Sweden; 3 Multidisciplinary Pain Centre, 8097 Uppsala University, Uppsala, Sweden; 4 Plastic & Oral and Maxillofacial Surgery, 8097 Uppsala University, Uppsala, Sweden; 5 Centre for Clinical Research, 8097 Uppsala University, Västerås, Sweden; 6 Department of Orofacial pain and jaw function, Faculty of Odontology, 5264 Malmö University, Malmö, Sweden

**Keywords:** S-Ketamine pain, pain, postoperative pain, pain management, intravenous, sedation, oral surgery

## Abstract

**Background:**

In most cases, a combination of paracetamol and ibuprofen are the optimal treatment for postoperative pain in third molar surgery. If stronger analgesia is required, opioids are traditionally administered. In day-case, surgery; however, opioids should be avoided. Thus, the anaesthetic agent S-ketamine in analgesic doses might be preferred.

**Methods:**

The study was designed as a randomized placebo-controlled double-blind clinical trial. The study enrolled healthy subjects according to the American Society of Anaesthesiologists classification; I or II (ASA), aged 18 to 44 years, with a body weight between 50 and 100 kg. The patients were randomized into three groups where two doses of S-ketamine were compared (high: 0.25 mg/kg or low: 0.125 mg/kg) with placebo (saline).

**Results:**

A primary outcome of the study was that VAS at 4 h postoperatively, showed no significant difference between the placebo and high-dose S-ketamine group or in the low-dose group. We found a significant difference between the groups for the first 24 h, with a lower VAS-score in the high-dose S-ketamine group. The time to when 50% had taken their first rescue medication was 12 min later in the high-dose ketamine group.

**Conclusions:**

Pre-emptive S-ketamine 0.25 mg/kg gave a global significant reduction of pain by VAS during the first 24 h postoperatively. The time from end of surgery to first rescue medication were longer in the high-dose ketamine group compared to both low-dose ketamine and placebo groups.

## A statement ‘Significance’, What’s already known about this topic?

• Ketamine is a versatile anaesthetic agent with useful analgesic properties in a wide range of pain management applications, acute as well as chronic.

### What does this study add?

• 0.25 mg/kg preoperative S-ketamine reduces postoperative pain VAS-score in third molar surgery significantly more than placebo or 0.125 mg/kg pre-emptive S-ketamine, during the first 24 h after surgery.
• 0.25 mg/kg preoperative S-ketamine increases time to first rescue medication in third molar surgery compared to placebo or 0.125 mg/kg pre-emptive S-ketamine.

## Introduction

Third molar surgery is one of the most common surgical procedures globally^1,2^ and causes moderate to severe postoperative pain.^3–5^ It is an often used and well established as a model in pain research.^6–8^ The clinical routine in third molar surgery is based solely on the use of local anaesthesia. Understanding that postoperative pain peaks approximately at the time when the local anaesthesia wears off, means that there is a need for ideas around the optimization of postoperative pain management.^3^ Ibuprofen alone or ibuprofen combined with paracetamol is commonly used and represents a way to manage the acute postoperative pain.^9^ In cases where ibuprofen and paracetamol are insufficient, opioids has been the next step to obtain pain relief. Opioids are valuable tools to manage moderate and severe postoperative pain, but are associated with side effects that make them ill-suited for day-case surgery, that is, hypotension and respiratory depression.^10–12^ There is a trend to reduce or eliminate the use of opioids in day-case surgery in general, which extends to oral and maxillofacial surgery practice.^13–17^

One alternative is the use of intraoperative ketamine, which reduces pain, respiratory and circulatory side effects as well as opioid use after surgery.^10,18^ In 1965 Domino et al first described CI-581, a derivative of phencyclidine^19^ and the first test of the drug in humans took place in 1964. CI-51 later become ketamine, a drug with analgesic and anaesthetic properties.^19,20^ Ketamine is primarily a general anaesthetic agent but is also widely used for analgesic purposes.^10,18^ Due to its strong analgesic effect and minimal impact on airway and respiration, ketamine is especially suited in trauma, prehospital practice and in circulatory unstable patients.^21,22^

Ketamine’s sympathomimetic effects lead to tachycardia and an increase in blood pressure. Common side effects of ketamine such as hallucinations and unpleasant dreams are reduced or eliminated through the use of benzodiazepines and in particular midazolam.^23,24^ Subsequently, because of the effect of benzodiazepine, the timing of ketamine administration should be coordinated with that of midazolam. Ketamine has a wide margin of safety and there are reports where children have been accidently overdosed five to 100 times the intended dose without any adverse outcome.^25,26^ At the same time, ketamine is coupled to a risk of addiction. Several harms stemming from chronic, addictive or recreational use of ketamine, such as ulcerative cystitis, neurological, cognitive and psychiatric impairment were reported.^26^ These factors may however not be relevant in a single dose clinical setting.

The aim of this study was to investigate if preoperative single dose intravenous (IV) S-ketamine has a role in day-case surgery of third molars related to pain experience and the need for complimentary analgesics.^15^

## Methods

The study was designed as a randomized placebo-controlled double-blind trial. The inclusion started in February 2017 and was completed in March 2022. The clinical part of the study suffered some delay due to the Covid-19 pandemic.

### Population

The study enrolled healthy individuals according to the American Society of Anaesthesiologists classification; I or II (ASA).^27,28^ Potential participants aged between 18 and 44, with a body weight between 50 and 100 kg were invited to participate in the study. The participants were selected from patients who had been, referred to the Oral and Maxillofacial Surgery clinic, at Falun County hospital (Sweden) for surgical removal of impacted lower third molar.

**Figure 1.**
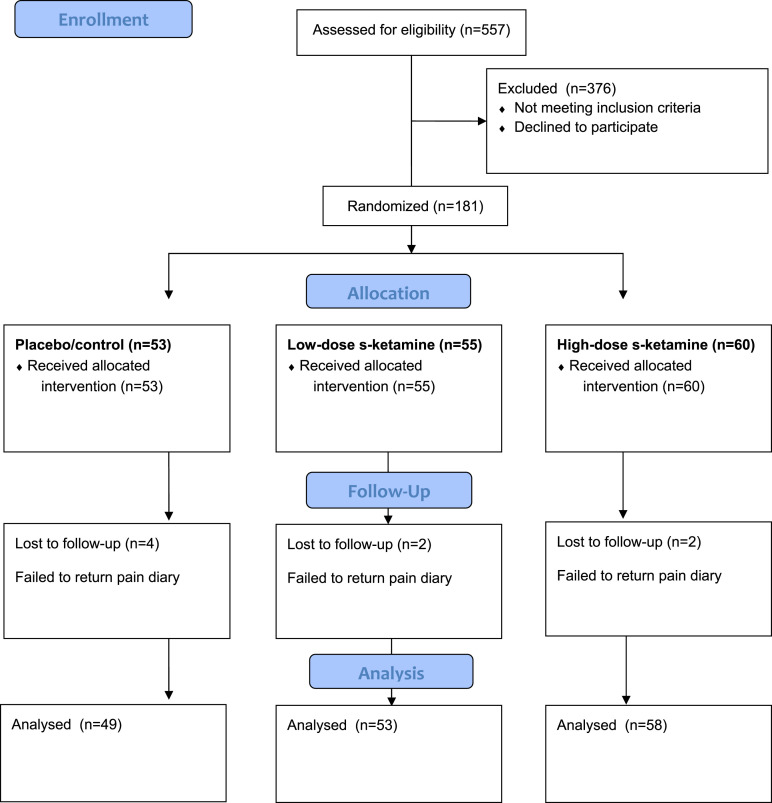
Flow of participants through the study.

The exclusion criteria was based on any of the following conditions: Potential participants were excluded from the study if they were receiving ongoing regular medication with analgesics, hypnotics, thyroid hormones, psychoactive drugs or MAO inhibitors. Potential participants were excluded from the study if they were diagnosed with or were currently undergoing, hypertension, heart failure, psychosis, epilepsy, hyperthyroidism, myasthenia gravis, glaucoma, verified sleep apnoea, diabetes, porphyria, pregnancy, breast-feeding, blood transmitted infections (such as HIV and hepatitis B and C) or if they had a known hypersensitivity to midazolam, ketamine, ibuprofen or local anaesthetics. Potential participants were excluded from the study if informed oral and written consent was not attained. A flow-chart of the study design is visualized in Figure 1 (Figure 1).

The subjects were randomized into three groups comparing two doses of S-ketamine, 0.25 mg/kg or 0.125 mg/kg, with placebo (saline). In a consecutive series, patients were offered participation in the study until at least 165 were included. The number of subjects was decided based on a power analysis.

We estimated the sample size necessary to detect a clinically meaningful mean difference of 15 mm in the VAS-score (0–100 mm) between high-dose ketamine and placebo at 4 hours post-surgery. From our previous observations, we expected a standard deviation of 26 mm, which for 80% power, a two-sided 5% significance level, and allowing for 15% attrition corresponded to 56 patients per intervention group.

### Randomization

A total of 168 subjects were randomized at a 1:1:1 allocation ratio to high-dose ketamine, low-dose ketamine or placebo, using stratified block randomization with a fixed block size of six within sex. In practice, the randomization sequence was generated through manual card shuffling, whereby two piles of six cards each were sequentially dealt and re-shuffled until completed assignment. The randomization sequence was stored at the local hospital’s pharmacy under the supervision of independent pharmacists, who also dispensed the study pharmaceuticals, which had an identical appearance to maintain concealment. Patients were allocated following eligibility criteria assessment, informed consent and completion of baseline measurements. All parties remained unaware of allocation until completion of the statistical analyses (Table 1).

**Table 1.** Demographic data and clinical characteristics of the study participants at baseline.

Baseline characteristics	Group		
	Placebo	Low-dose s-ketamine	High-dose s-ketamine
	Saline solution	0.125 mg/kg	0.25 mg/kg
No.	53	55	60
Age, mean (SD), y	30 (6.7)	29.5 (6.7)	28 (6.7)
Weight, mean (SD), kg	74.8 (13.5)	74.9 (14.6)	72.8 (13.0)
Height, mean (SD), cm	171.3 (8.4)	171.6 (8.7)	171.6 (8.8)
BMI, mean (SD)	25.4 (4.1)	25.5 (8.7)	24.7 (4.1)
Sex			
Male	17 (32%)	17 (31%)	19 (32%)
Female	36 (68%)	38 (69%)	41 (68%)

### Clinical procedure and intervention

#### Logistics

A list of serial numbers and randomized groups affiliation was produced, which was accessible to the pharmacy's manufacturing staff and closed to all staff performing the clinical part of the trial. The clinical trial leader informed the pharmacy which serial numbers were in turn for treatment and the pharmacy prepared filled syringes with the investigational drug in accordance with the randomized group affiliation. Preparation took place in the pharmacy's manufacturing unit. The syringe containing the test drug was marked with Code/serial number, shelf life/expiration date, time of manufacture, patient name, id-number and EudraCT number. The prepared investigation drug was delivered to the clinic maximum 24 h in advance.

#### Blood sample

A peripheral venous catheter was placed in a superficial vein of the subject’s forearm or on the back of the hand. The peripheral venous catheter was used for drug administration and for blood sampling (Table 2).

**Table 2.** Routine clinical chemistry of liver and kidney function at baseline.

Baseline characteristics		Group		
		Placebo	Low-dose s-ketamine	High-dose s-ketamine
		Saline solution	0.125 mg/kg	0.25 mg/kg
Albumin (μkat/L)				
	mean(SD)	41.7 (2.9)	41.6 (3.0)	41.8 (2.9)
	min/max	36/49	34/50	35/50
ALT (μkat/L)				
	mean(SD)	0.6 (0.2)	0.6 (0.2)	0.5 (0.1)
	min/max	0.3/1.8	0.3/1.8	0.2/0.8
AST (μkat/L)				
	mean(SD)	0.5 (0.1)	0.5 (0.1)	0.5 (0.05)
	min/max	0.3/1.4	0.3/1.0	0.3/0.6
Creatinine (μmol/L)				
	mean(SD)	67.5 (11.4)	67.8 (12.4)	68.9 (11.9)
	min/max	46/93	48/102	46/98
eGFR [ml/min/1.73 m^2^]				
	mean(SD)	93.1 (7.2)	93.1 (8.4)	92.7 (6.5)
	min/max	75/100	73/100	78/100

#### Sedation

All participants in every group were sedated with midazolam intravenously to a defined end point: Ptosis and/or dysarthria. The oral and maxillofacial surgeon (LBE) and an independent observer assessed sedation depth at the endpoint according to the Observer's Assessment of Alertness/Sedation Scale (OAA/S scale).^29–31^ The rating was from 1 (deep sleep) to 5 (fully awake). Four variables were evaluated: responsiveness (1 to 5), speech (2 to 5) facial expression (3 to 5) and eyes (3 to 5). The lowest of all values from the four variables were noted. The scale is regarded as valid and reliable in relation to VAS, the University of Michigan sedation scale (UMSS), and electroencephalogram bispectral index analysis (EMG-BI).^29–31^

#### Investigational drugs

In the literature, we found a broad spectrum of doses, 0.075 mg/kg to 1 mg/kg as bolus, but there appears to be no consensus as to which S-ketamine dose should be used in postoperative analgesia.^18^ We thus used two different doses to enable comparisons. We looked for doses likely to give analgesic effect but not cause general anaesthesia. One American consensus guidelines stated that the common subanesthetic dose of ketamine used in clinical practice is IV 0.3–0.5 mg/kg bolus with or without an infusion.^32^ The recommended dose to induce anaesthesia is 0.5 to 1 mg/kg IV or 2 to 4 mg/kg intramuscular (IM).^33,34^ We chose to give the ketamine prior to surgical incision to enable a standardized protocol. An injection at the end of or after surgery would have been less coordinated with the midazolam effect. The drugs were administered as follows to the three groups:

(1) Placebo group: Sodium Chloride solution (9 mg/ml) 0.2 mL/kg slow intravenous injection (2 mL/min).
(2) Low-Dose Ketamine group: S-Ketamine (0.125 mg/kg body weight).
(0.625 mg/ml x 0.2 ml/kg) slow intravenous injection (2 ml/min).
(3) High-Dose Ketamine group: S-Ketamine (0.25 mg/kg body weight).
(1.25 mg/ml x 0.2 mL/kg) slow intravenous injection (2 mL/min).

The test drug was injected using a syringe pump (Alaris CC, Cardinal Health, Rolle, Switzerland) at a rate of 120 mL/h (2 mL/min) with volume accuracy ±2%. One infusion set (Alaris Medical System Ref MFX2299 E Impromediform GmbH, Lüdenscheld, Germany) was coupled between the syringe and a three-way connection, which in turn was connected to a peripheral venous catheter (Venflon® 18G 1.3 × 32 mm). The test drug was administered as a pre-emptive single dose immediately after the sedation. Total injection time varied between 5 and 10 min depending on the patient’s body weight of 50 to 100 kg.

The surgical procedure was performed under local anaesthesia (LA) Xylocaine adrenaline 20 mg/ml, four ampoules of 1.8 mL, supplemented if necessary to achieve intraoperative pain relief. The LA was inferior alveolar nerve block in combination with buccal infiltration. The affected lower third molar was removed according to an established surgical procedure, which started with a mucoperiosteal incision at a 45° angle from the distal buccal part of the tooth and a small buccal flap was mobilized. After bone removal around the root, the tooth was luxated and sectioned at the root furcation if needed and the parts were removed. The wound was inspected for haemostasis, irrigated with saline and the incision closed with a resorbable suture.

#### Perioperative monitoring of the patients

The patients were monitored with pulse oximetry continuously and non-invasive blood pressure measurement every fifth minute using an automatic patient monitor (Dash 5000, General Electric Company). The respiratory rate was registered manually during 1 minute at four occasions before and during the surgery. The antidote for benzodiazepines (flumazenil) was on hand. Direct access to supplemental oxygen in case of desaturations or assisted breathing as well as suction catheters for cleaning of the upper airways were available, tableside. After the surgery, all patients stayed for recovery time and before discharge, the patients were given oral and written postoperative information. Every participant was given a box with 30 rescue pills of Ibuprofen (Ibumetin®) in doses of 400 mg, to be taken up to four times a day, administered as needed by the patient.

#### Outcome measurements

Primary outcome was VAS ∆ = VAS pain 4 h - VAS pain preoperatively (mm)^35–37^ and The secondary outcome variables were time to first rescue medication (minutes), number of rescue pills first 24 h, use of complementary analgesics, and safety, which will be separately reported in detail.

The patients rated their spontaneous pain intensity on eight occasions during the first 24 h (immediately before and after surgery, 2, 3, 4, 6, 8, 24 h after end of surgery) using a 100 mm scale, VAS. The endpoints of the scale were marked ‘no pain’ and ‘worst pain imaginable’.^38^ Pain during function such as chewing or swallowing was not measured. Analgesics consumption was described as the time to first rescue medication and total analgesic consumption for the first 24 h, as well as use of other analgesics than those provided in the study.^38^

### Statistics

#### Analysis

Data were analysed according to the intention-to-treat principle, where participants are coded to their allocation status. In the primary analysis, the VAS Δ-score at 4 hours post-surgery was compared with the Mann–Whitney U test in a hierarchical procedure of predetermined order to retain the nominal 5% significance level^1^: high-dose ketamine and placebo and^2^ low-dose ketamine and placebo (R v4.2.2). Four secondary analyses were also conducted. First, the VAS-score was compared between the intervention groups per time point in a linear mixed model for repeated measures (nlme v3.1–160 in R v4.2.2). The model included time as a factor, the interaction between time and intervention groups for all time points except baseline, and the stratification factor ‘sex’ as fixed effects. To account for dependency within the patient, it included a patient-specific random intercept and an autoregressive correlation structure for the repeated measures. Based on this model the marginal means per time point and intervention group as well as globally across time points was estimated (emmeans v1.8.2 in R v4.2.2). Second, time to first pain medication was computed with the Kaplan–Meier estimator and compared between high-dose ketamine and placebo using the log-rank test (survival v3.4.0 in R v4.2.2). Finally, the total number of pain medication pills were compared between high-dose ketamine and placebo using the Mann–Whitney U test (R v4.2.2). Besides the aforementioned analyses, adverse effects were described, including saturation during surgery and the frequency of hallucinations, nightmares and nausea, post-surgery.

#### Ethical considerations

The difference from conventional clinical routine treatment was the venous blood sampling, which implied a further invasive step. However, in relation to local anaesthesia and surgery, this was considered negligible or of minor importance.

The Swedish Ethical Review Authority revised and approved the study protocol (Dnr 2015/378). The Swedish Medical Products Agency (MPA) granted us permission for this study regarding clinical drug testing on humans (Dnr 5.1-2016-48439). The study was registered in European Union Regulating Authorities Clinical Trials Database (EudraCT) with number: 2014-004235-39 and was registered in CinicalTrials.gov, ID: NCT04459377.

Permission to set up a sample collection at the local biobank was obtained (sample ID 873-2014-004-235-39). Further, we obtained verbal and written informed consent from each patient prior to initiation of any study procedure. In all clinical and administrative aspects, Good Clinical Practice (GCP), General Data Protection Regulation (GDPR) and the Helsinki Declaration was followed.

S-Ketamine is a registered drug and thus already well documented. This study was a phase IV clinical trial and has therefore undergone a rigorous application procedure to obtain a permit from MPA for clinical drug testing in human. In this process a general safety assessment was carried out, but a plan was also put in place in order to detail how any adverse events (AE), serious adverse events (SAE) or suspected unexpected serious adverse reactions (SUSAR) would be handled. Grounds for detection were spontaneous report, observation including laboratory reports, and when needed active questioning. Furthermore, the principal investigator (PI) and the sponsors’ responsibilities were described. Possible actions within the trial could be to break the blinding code or to stop the trial. The ultimate protection of the participants’ health was furthermore to provide any medical attention needed. In the trials, specific standard operating procedure (SOP) contact information to related authorities was available in case of any AE, SAE or SUSAR were to be reported.

Since the drug is registered, the trial leader (LBE) had already acquired clinical experience of the drug in clinical use. Although these previous treatments have not been recorded and studied systematically, no obvious side effects were noted.

Nevertheless, there are several known side effects to ketamine use: dreams, nightmares, vertigo, restlessness, blurred vision, palpitations, increased blood pressure, irregular or slow pulse, decreased blood pressure, effects on breathing, nausea, vomiting and increased salivation. Others, such as increased body movements (e.g. muscle twitching that may resemble seizures), increased eye movements, double vision, increased intraocular pressure, skin rashes, pain and/or redness at the injection site, allergic reactions, hallucinations, depression, anxiety and confusion have also been reported.^39,40^

#### Monitoring of the project

An independent external person performed monitoring at four occasions during the study period: the first visit before inclusion, the second and third visit during ongoing inclusion and the last visit soon after the inclusion was completed.

## Results

### Drop out

The inclusion took place on the day of surgery. Due to this study design, there was no drop out. Nevertheless, there were some missing data but only a few.

### Duration of the surgery

Median duration of surgery was 15 min with no significant difference between the groups.

### Self-reported spontaneous pain by VAS

**Figure 2.**
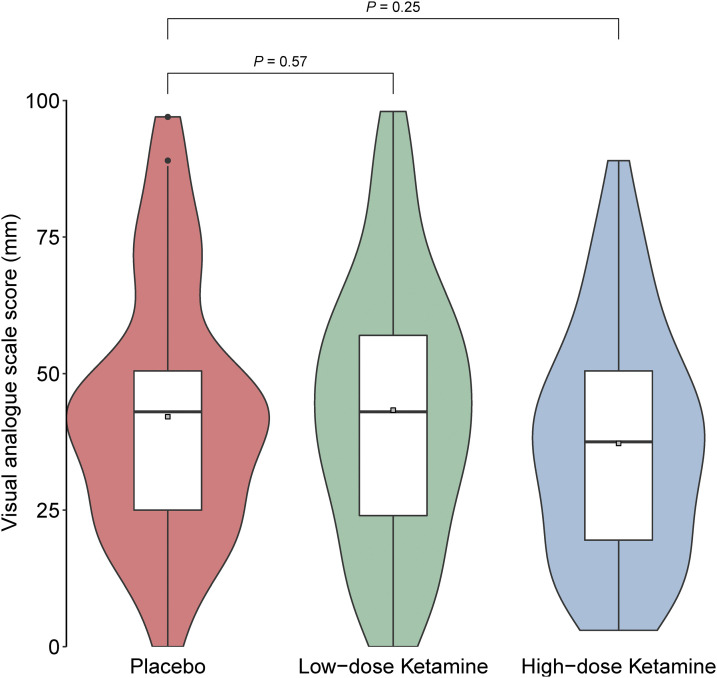
Combined box and violin plot. Violin plot distribution of VAS Δ-score for pain at primary end-point (4h post operatively), no significant difference between groups. (Red: Placebo; green: low-dose S-ketamine; blue: high-dose S-ketamine.) Box plot Grey square dot = mean; horizontal line = median; top of box = third quartile; bottom of box = first quartile; vertical line = upper and lower adjacent value.

**Figure 3.**
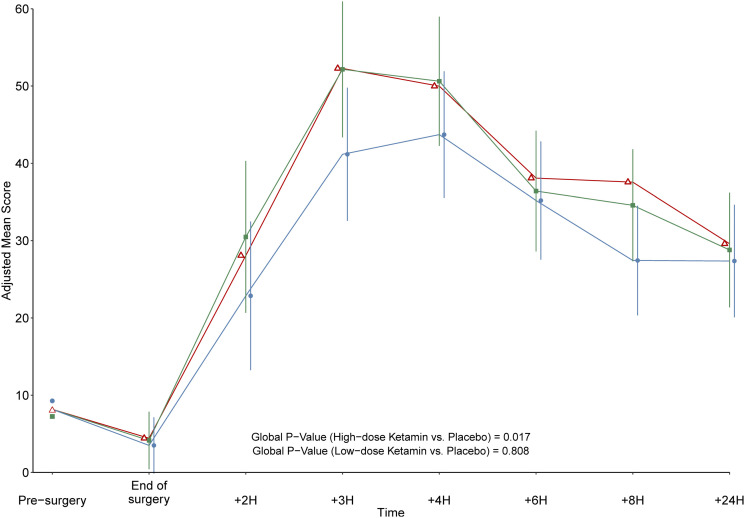
VAS pain the first 24 h. Data markers and lines represents the mean score for each intervention group. They have been adjusted for the baseline values with the exception for time zero where the data markers indicate the actual baseline scores. Error bars denote the 95% CI of the difference between S-ketamine and placebo at each time point, centred on the mean values of the S-ketamine groups. When the 95% CI overlaps with the mean line of the placebo group, the *p* value of the difference is greater than 0.05. (Square: low-dose S-ketamine, circle: high-dose S-ketamine, triangle: placebo).

Primary outcome, VAS Δ at 4 h postoperatively, showed no significant difference between the placebo and high-dose ketamine group [Figure 2]. We found a significant difference in mean VAS between the groups for the period 2–24 h, with lower VAS-score in the high-dose S-ketamine group [Figure 3].

### Time to rescue medication

**Figure 4.**
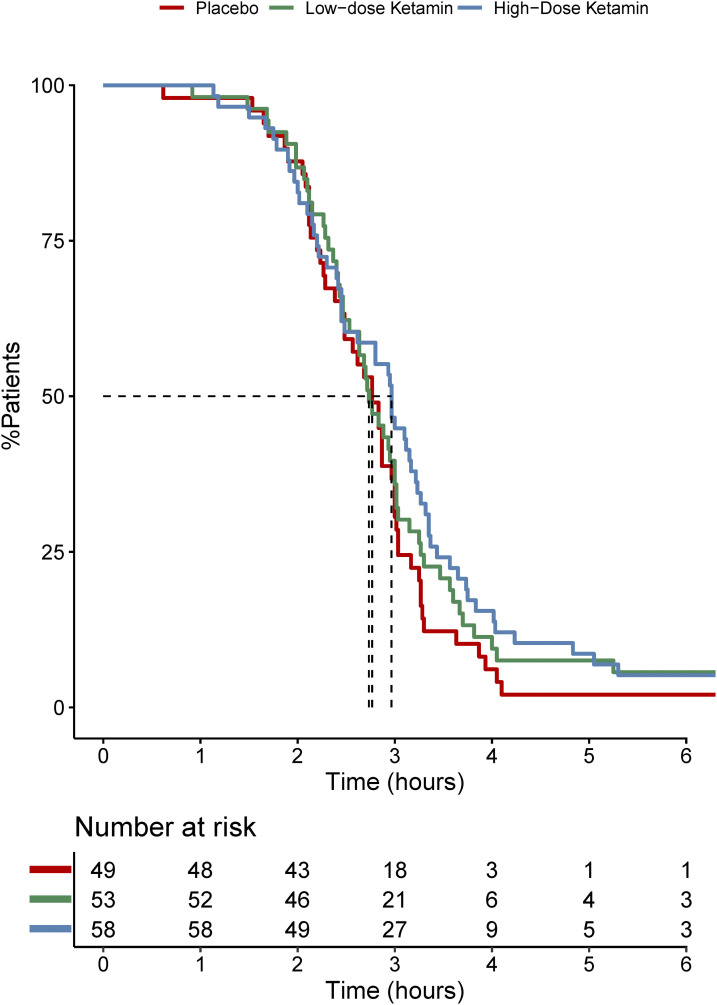
Kaplan–Meier curve of time to first rescue pill. High-dose group are 12 min later than low-dose and placebo to 50% of patients have taken first pill (*n*.s.). Dotted vertical line show median survival time per group.

The median time to first rescue medication was 12 min longer in the high-dose S-ketamine group [Figure 4]. The total amount of rescue pills during the first 24 h did not differ between the groups. The use of additional analgesics was similar between the groups.

### Side effects

Hallucinations and nightmares were relatively few and equally distributed in all groups. Nausea was more frequent in the high-dose S-ketamine group, but still in small numbers (12 of 60 subjects).

In a randomized design such as ours, there are no need for comparing baseline data between the groups.^41^ Nevertheless, we can state that the difference between the groups were minimal and non-significant regarding demographics as well as pain score data [Table 1].

## Discussion

Our study showed a reduction in global mean pain during the first 24 h after surgery for those patients who received high-dose S-ketamine (0.25 mg/kg). No such effect was shown in the low-dose S-ketamine group.

More relevant than a statistical pain reduction is a clinical relevant pain reduction. The problem is to decide whether the change or difference is clinically relevant. Presently, there seems to be no consensus about the size of clinical relevant change of VAS in acute postoperative pain clientele. In chronic pain, a 30% reduction of pain from baseline is considered clinically relevant when measured on a VAS or NRS (numeric rating) scale.^38,42^ A change by 18 out of 100 mm is considered a clinical relevant difference reported in a publication dealing with cancer break though pain^42^. In an Australian study of acute pain from 1996, the authors found that 9 mm was the minimum VAS score difference of clinical significance.^43^ For this study, we decided to use 15/100 mm as a clinically relevant change in pain VAS score as was used in a previous study from our group.^44^ With this in mind we find our results clinically relevant.

Ketamine was first registered on the Swedish market in 1973 by Pfizer as Ketalar ®. The s-isomer reached the Swedish market later in 2013, and was registered by Pfizer as Ketanest ®. Despite many years having passed since its introduction to the market, ketamine is not widely studied in oral and maxillofacial surgery applications such as perioperative analgesia in day-case practice. In our literature search from 2009 to the beginning of 2019, we found 454 articles of which only five were relevant to our project^45–49^ and only two of those covered the IV route for administration.^45,47^ Both studies were small in numbers *n* = 50 and *n* = 36, and only one of the two was placebo controlled.^45^ The other study used remifentanil as control.^47^ Even as a base medication equal to all participants, the combination of several analgesic, anaesthetic and sedative drugs in a study with few subjects in groups makes it challenging to know what causes a possible positive effect. In this regard, our design seems robust and sufficiently large to test our hypotheses. The sample size did fit our intention to evaluate S-ketamine’s role in some aspects of the postoperative pain experience, but may not be sufficient to find unknown or even rare side effects where a larger sample would have been needed.

The willingness to participate in clinical trials has been analysed regarding several factors in several studies.^50,51^ A skewed sex distribution was found in our material, where two thirds of the participants were women, which contrasts with literature that describes a greater challenge to enrol female participants^52^ or in some articles no difference due to sex.^53^ This imbalance is partly explained by a slightly greater number of females diagnosed with impacted teeth and a slight majority of women undergoing surgical removal of teeth at our clinic. The sex imbalance was equal in all three groups. Though the statistical method compensated for the imbalance in the sex of the participants, this does not impact the internal validity of the results.

In this study, we found a lower global VAS-score and a small delay to first rescue medication in the high-dose S-ketamine group. Reported in a Cochrane review, a larger mean delay until first request for rescue analgesia or activating of patient controlled analgesia (PCA) (54 min) was described.^18^

Our observations of improved pain control may indicate that a slightly higher dose ketamine is needed. Although our findings might be too small for clinical relevance, they may be valuable for selected groups, i.e., patients with known problem with pain management after previous minor surgery or patients that should avoid opioids for some reason. One such reason is day-case surgery patients who require opioids. Patients with pre-existing chronic pain undergoing third molar surgery may also benefit from ketamine reinforced perioperative analgesia. In these instances, ketamine may offer a therapeutic option that alleviates patients’ perioperative pain.^10,54^ In multiple strategies for perioperative pain management S-ketamine has advantages in comparison with opioids. S-ketamine is associated with less emetic side effects as well as maintaining blood pressure, pulse, breathing and a free airway compared to opioids. There are limitations to this study, as S-ketamine in single dose is quite short lasting, while postoperative pain after third molar surgery remains for several hours even days. A repeated or continuous administration of S-ketamine may be preferable to optimize the duration of the effect. At the same time, there is an aspiration to minimize the time span when the patient is under the influence of the S-ketamine, which in turn might delay the point when the patient recovers street fitness and can be discharged.

## Conclusions

Preoperative S-ketamine 0.25 mg/kg gave a global statistically significant reduction of spontaneous pain by VAS during the first 24 h postoperatively. Preoperative S-ketamine 0.125 and 0.25 mg/kg gave no significant pain reduction at 4 h postoperative. The time from end of surgery to first rescue medication was slightly longer in the 0.25 mg/kg group compared to both low-dose S-ketamine and placebo.
